# Aspirations and Compromises: Changes in Homestead Space Relations of the Extreme Poor after Disaster

**DOI:** 10.1371/currents.RRN1280

**Published:** 2011-11-01

**Authors:** Tahmina Rahman

**Affiliations:** Architect, associated with reconstruction activities and researches, postgraduate programs in disaster management, brac university, dhaka, bangladesh

## Abstract

Background: Construction of houses in homesteads and their settings occur in the context of traditional perceptions and practices in the rural culture of Bangladesh. Functional spaces inside and around the house are produced according to need over time. Inhabitants construct their houses with locally available resources and knowledge. After devastating disasters houses are delivered as products by the development agencies to quickly cater to the needs of the sufferers. The extreme poor are the receivers and inhabitants of these new houses, which can cause significant changes in the physical and environmental characteristics of the neighborhood. In this regard the building and dwelling values of the inhabitants in relation with these houses may be changed or lost. But these values are otherwise inherent characters of the rural houses in the habitations that are shaped by the aspirations of the dwellers.

Methods and Findings: This paper investigates how relief houses serve the needs of the extreme poor after disasters and how these houses gradually blend with the surrounding environment matching with the aspirations of the inhabitants. The methodology followed was observation of the backgrounds of the pre and post disaster situations, focus group discussions, drawings sessions and interviews with the inhabitants, craftsmen and locals, use of secondary sources, and visits to the houses during and after construction to understand the techniques and space value.

Conclusions: The present practice of distribution of relief houses without involvement of the owners either in the information sharing or building processes and without understanding owners’ perceptions about dwellings, may compromise the compatibility and hence the sustainability of relief houses. Hence, houses may only be used as temporary or transitional shelters to sustain life in the disaster phase, and will not be used as “houses” long term.

## Introduction

 Houses as a relief item for the extreme poor have become a major reconstruction practice in the southwestern region of Bangladesh especially after the devastating cyclone Sidr of November, 2007. Natural disasters bring significant changes to habitations and the physical environment. But in spite of the negative consequences, these massive destructions may be perceived as catalyst of changes that can promote the practice of aspirations of the communities associated with it.

Aspirations concerning house constructions and habitations are reflected in the reconstruction phase. But the aspirations are perceived in different directions by the different stakeholders. After substantial destruction, involvement of development organizations in rapid house constructions became necessary. It has been observed that “houses” are given to the recipients only as relief of basic necessity. The morphology of these houses, materials used or spaces created did not always reflect the lifestyle or the vernacular patterns of the inhabitants. The building as a product (noun) and building as an activity (verb) often failed to support the dwelling as a quality (adjective). “…dwelling would in any case be the end that presides over all buildings. Dwelling and building are related as ends and means. However, as long as this is all we have in mind, we take dwelling and building as two separate activities, an idea that has something correct in it. Yet at the same time by the means-end schema we block our view of the essential relations” (Heidegger, 1971)[1]. The quality of dwelling of man as builder and inhabitant on his habitation and his aspirations could not always be perceived clearly in the relief houses.

In this paper I aim to explore the qualities of these relief houses as structures and how they integrate into the homesteads and the settlement.

## Objective and Methodology

The objective of the paper is to investigate how relief houses serve the needs of the very poor after disasters, in the context of disaster relief and in addition to understand how is used, and how these relief houses gradually blend with the surrounding environment and match with the aspirations of the inhabitants.

The methodology was observation to understand the backgrounds of the pre and post disaster situations ; focus group discussions; drawings sessions and interviews with the inhabitants, craftsmen and locals; use of secondary sources; visits to the houses during the construction phase and after to understand the techniques used and how the spaces provided by these houses provide value and match with the aspirations of the inhabitants.

## The Study Setting

The study was carried out in some villages at Sarankhola upazilla of Bagerhat district in Bangladesh during the reconstruction period after cyclone Sidr in 2007, during the monsoon of 2008 (Rahman, T, 2008)[2]. Bagerhat was declared as one of the four worst affected districts in the cyclone with 118,899 houses totally and 130,675 partially damaged (MoFDM, 2008)[3]. This was reported to be among the maximum (GoB, 2008)[4]. Sarankhola suffered most of the damage including casualty, house and other infrastructural damages.

Relief houses distributed at the selected region by BRAC (with two of its models), Islamic Development Bank and DanChurchAid were selected as case studies. Data was collected from the field from the users and craftsmen with limited number of open ended interviews and transect walk.  

## Background

 The extreme poor are the ones who are unable to meet the minimum standards of basic needs, do not have a secure source of income, are obliged to spend most of the earning on food but fail to fulfill the minimum calorie intake, and are in poor health that negatively affects their condition and resources (Abed, F, H, in Matin, I and Walker, S, 2004)[5].

The extreme poor live in the most basic form of shelter with materials mostly natural and collected from the surroundings where the quality of space is constantly changing as a result of its repair and renewal (Kabir, K, H and Mallick, F, H, 2006)[6]. This is a normal process in the development with decay and regeneration. Spaces frequently change their quality and function in this approach. After a disaster, on the contrary, the functional and spatial characters of habitation and the associated spaces are lost to some extent which needs to be readdressed soon for the sake of the habitants.

Aspirations concerning house construction of the inhabitants are reflected in the indigenous practices of a locality, the different values and customs, space use patterns, materials and methods used (Baqee, 1998)[7]. Aspirations of development organizations concerned with relief houses can be perceived through the approaches, target groups and extents exercised in the field (IFRC, 2007, UN-HABITAT and IFRC, 2010)[8] [9]. As the scale of rebuilding after major disasters is massive and needs to be completed within a short time, the possibilities to blend the potentialities of the both stake holders are often not explored fully. This creates gaps in the process of sustainability (O’Brien, D, Ahmed, I and Hes, D, 2008)[10].

It has been observed from different field experiences that the recipients know best what they need (Oxfam America, 2010)[11]. But it is often that the relief operations are driven as such that the specific needs of the recipients cannot be addressed in the package either in the “product” or the “process” manner. 

## The Settlement, Homestead and Inhabitants

 The settlement pattern in the studied region is dispersed because of the geographical setting and livelihood pattern. It is surrounded by lowlands submerged in water most of the year, except in winter. The dispersion may also occur as the inhabitants do not have large amount of common properties to share or guard. Higher density and squatter-like linear settlements occur near the embankments because of scarcity of high land that is free from the risk of flooding. Livelihood is share cropping, collection of shrimp fries from lowlands, and seasonal collection of forest materials from the Sundarbans, the mangrove forest.

The limited road network connects only a small portion of the locality. Most of the roads are unpaved. During focus group discussion and drawing the inhabitants of a village drew a map of the paved road of the locality showing connections of the houses of affluent persons to present an idea of the built environment (Fig: 3). The main transport in the paved road is rickshaw van.

**Figure:**
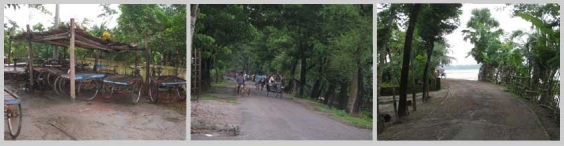
*Figure 1: Rickshaw vans, the main transport and paved roads in the community *

**Figure:**
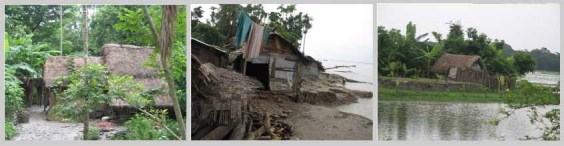
*Figure 2: Houses with natural materials*

**Figure:**
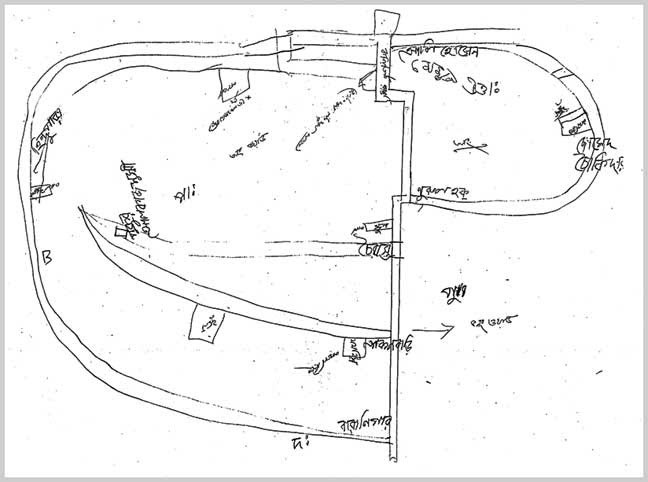
*Figure 3: Diagram from Focus Group Drawing and Discussion showing road network*

Homesteads are on plinths made from mud gathered from surrounding. It is sticky and gets hard when dry. The house is comprised of one large structure with rooms inside it, it may be one or two storied. Only toilet and kitchen are outside this main house structure. Mostly locally available primary and natural materials are used for house constructions. Wood is abundantly used both as frame and walling materials that is gathered from the harvest of social forestry and from the forest. Thatch materials, palm fronds, sun grass and nipa leaves (from nipa palm, grows in mangrove environment) are extensively used as filler material and for roofing by the poor.

Houses of the extreme poor have one or two rooms under a common roof. Hipped roof is preferred as it gets less damage in strong wind (Agrawal, A, 2007)[12]. Verandas are important in part for multipurpose uses like social gathering, and for keeping goats and chickens. Verandas can be covered to be used as extra room.  This semi covered space is called pashchati in local tongue. The roof of the veranda is structurally separate from the roof of the main house over the pashcahti. These usually do not share same angles. It has the best record of wind resistance in cyclone (Seraj, S, M and Ahmed, K, I, 2004)[13].

Plinth height is low. This may be a result of people not digging enough ponds and thus not using the excavated earth for plinth construction. Ponds are not common in the habitations in this region like the other regions of the country as pond water gets saline if it is at level with the ground water table.

Grain and other valuables are stored inside the houses on platform over the head space. In this way it remains safe in case there is flooding in high tide or storm surge.

Columns are not embedded inside the mud plinth with a footing, but these remain free standing over burnt clay plates on the edge of the plinth. It is traditionally constructed in this manner. So if the houses are carried away in strong current in a storm, the structure would have minimum damage and can be put back over the plinth again.

**Figure:**
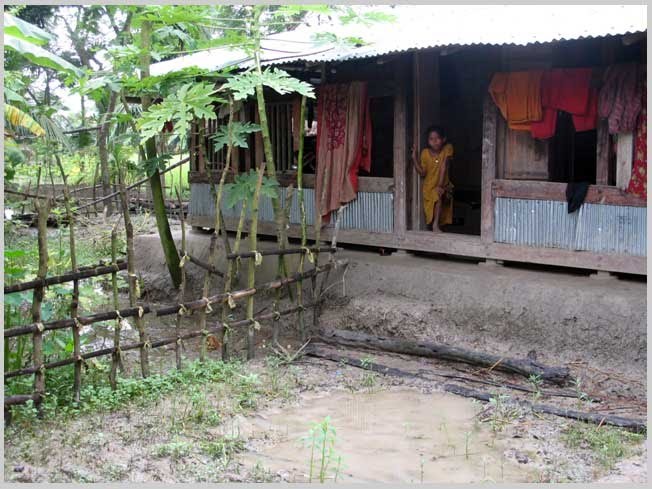
*Figure 4: wooden column in a traditional house*

The physical environment is intensely green. Timber and fruit trees are much appreciated as homestead plants for promotion of social forestry. The fabric of the settlements is natural because of house construction materials being gathered from nature and the distinctive green surroundings. This texture changes through the repair and maintenance activities but these are also in harmony with the seasonal changes. 

**Figure:**
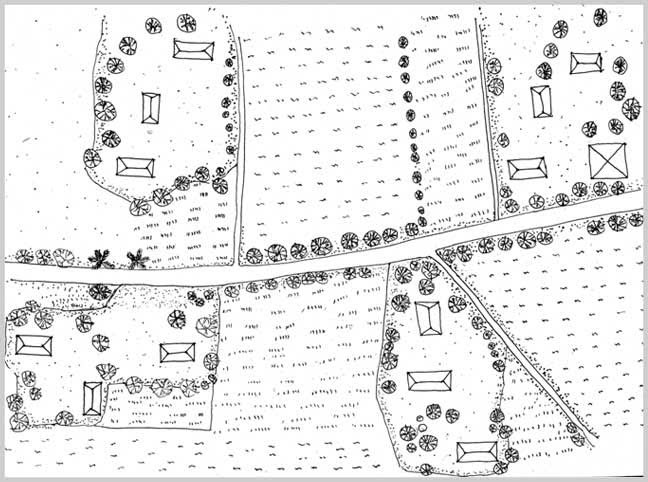
*Figure 5: Homestead planning in the habitat*

 In the natural settlement, neighbors are isolated. Social customs do not encourage the females to participate in outdoor activities like farming or fishing. Moreover they are occasionally obliged to live without the male guardians taking care of the family during November to February, the forest materials collecting season as the males are often in the forest then. But females in groups go far to fetch drinking water as many places lack services to distribute drinking water such as tube wells and community ponds. Tube wells are not popular because of the contamination of salinity in the water table.

## Case Studies          

 Apart from damage to life and properties, many trees were destroyed in the cyclone (GoB, 2008)[4]. Homestead mounds were damaged by surge water; the landscape remained unfit for rebuilding for a long time. Destruction of the Sundarbans and the prohibition on collection of forest materials for the forest to re-grow (The Daily Star, 2007)[14] added to the scarcity of house construction materials.

As property and livelihood was exceedingly damaged, large scale interventions in reconstructions became necessary. The development organizations involved in reconstruction chose model designs for house structures and industry produced materials for building construction.

In three of these case studies model houses designed by consultants were provided as grants except the DanChurchAid one, in which the owners made their houses with a limited amount of money and C I (corrugated iron) sheet received as grants for house construction. Quality control was facilitated by the providing NGOs or their local partners. It was agreed in the Government of Bangladesh Shelter Recovery Strategy (Shelter Coordination Group, 2007)[15] that shelter assistance would be shifted from providing houses to the affected families to involving the community in decisions and actions helping them in better coping ability and resilience.

 Some of the features of these houses are described in the table:

**Table:** 

**BRAC House**		**Islamic Development Bank House**	**DanChurchAid House**
**Hipped roofed house**	**Gabled roof house**		
Construction cost was sixty five thousand taka, this included four thousand taka as labor cost.	This was an earlier model of BRAC relief house. It was distributed before the new model with better wind resistance was introduced.		This was implemented through Dustho Shasthya Kendra (DSK). Twenty thousand taka and eighteen bundles of C I sheets were provided as relief package for house construction.
It had a room of fifteen feet by ten feet, with a veranda of fifteen feet by five feet at the front.	Floor areas of both of these houses were same.	It had a main room with an extended portion, but this could not be defined as a verandah or serve as one.	There was no set house model to follow. Recipients had freedom to choose size, form and materials according to need and also add own resources.
It had a hipped roof over the main house, and a lean to roof over the verandah, both of C I sheet. These two roofs were separate entities with separate timber frames.	It had a gabled roof over the main room and a lean to roof over the verandah. The verandah roof was an extension of the main roof sharing the same frame and angle.	It had a gabled roof with an addition lean to extension. These two roofs were separate from one another. The eave of the roof at the front was extended.	
It had four RCC pillars of four inch by four inch thickness and ten feet height at the corners. These were embedded three feet below the plinth, and tied to a ten inch wide RCC base with two inch long two metal clamps, screws and nuts. These were tied with the timber roof frame with twisted metal clamps, screws, nuts and metal wires.	RCC main pillars with reinforced base, additional wooden pillars, bamboo mat walls, wood frames, C I sheet roof, and metal wires and pegs were used.	Wood frame with C I sheet were used in walls and roof. Wood was used extensively.	Wood, bamboo, betel nut trunk were used in frame, iron pegs were used in joints. Frames of the structure rested directly on the ground without any footing.
It had six wooden pillars with metal clamps inside the house. Wooden horizontal frames were used in walls. The verandah at the front had four wooden pillars.		All the wooden posts were of same thickness and not embedded into the plinth. Only the four main posts were embedded inside the plinth.	
Mud plinth had low brick boundary walls and brick steps at the front.	Brick boundary and plinth heightening was done later.	Mud plinth had brick plastered boundary around it with steps at front. The plinth was higher compared to the other case studies.	
Cross bracings of wood was used on the outer walls.	Wooden frame with vertical and horizontal members were used instead of cross bracings.		The recipients constructed the houses without any craftsman’s help. It lacked improved techniques like cross bracings and metal clamps.
It had one door and two windows at the front.		It had one door and two windows at the front and two additional windows at sides of the main room.	
A platform above the head height provided a storing space inside the house.			
Walls were of bamboo mat treated with locally made oil varnish *(maitta tel).*		The full structure had enclosures of C I sheet from all sides and resembled like a box.	Many materials were reused, for example wooden walls of the old houses.

**Figure:**
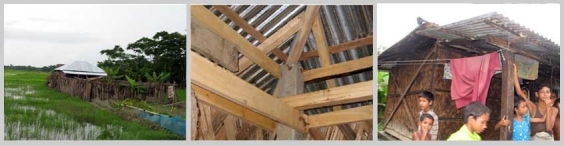

**Figure:**
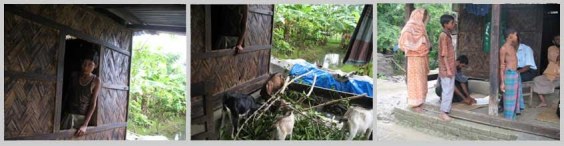
*Figure 6: Hipped roofed relief house by BRAC*

**Figure:**
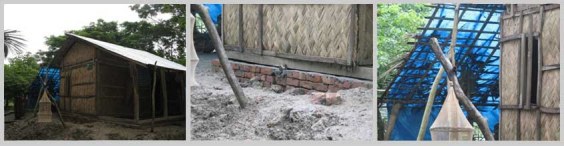
*Figure 7: Gabled roofed relief house by BRAC*

**Figure:**
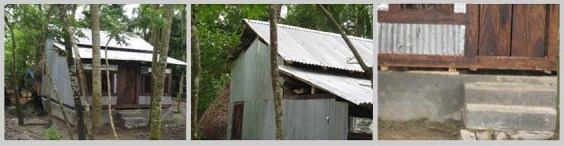
*Figure 8: Relief house by Islamic Development Bank*

**Figure:**
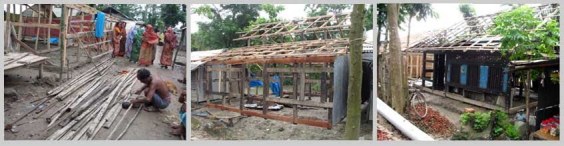
*Figure 9: House constructions from relief of DanChurchAid*

## Findings from User Aspirations and Needs

 The relief houses used materials and techniques many of which especially C I sheet, cross bracings, RCC (reinforced cement concrete) columns added to the aspiration of the inhabitants. According to their perception, they could not previously have afforded these materials and these techniques would not have been within their capacity. The disaster had presented them a chance to benefit from these. In their view these materials and techniques would reduce the repair cost and enhance their status in the society as they would not have to construct with leaf materials, which are considered fragile. Furthermore, they did not have the capacity and capital to build houses of such condition at this short time; it would have taken much more time to reach this position.

The houses introduced some new affordable and low cost ideas and techniques in the community that was highly appreciated by the users and craftsmen, e.g. the cross bracing. It was also well understood that metal angles and clamps in the joints and metal screws with bolts instead of only metal pegs make a structure much stronger and durable. RCC pillars with strong anchoring at the base prevent the house against getting uprooted and thus become more resistant to strong wind.  The gradual scarcity and incompatible standard of natural materials had been inclining people to construct with factory produced materials. Introduction of these affordable techniques by intervention through relief houses has the possibility of improving the construction methods in the locality.

Introduction of brick walls around the mud plinths was a new approach. It was observed that the inhabitants used bricks to cover portions of their yard especially the pedestrian routes to function better during the rainy days. But brick construction around the mud plinth reduces rain damage and thus increases stability (Ahmed, K, I, 2005)[16]. It is often that the full house structure needs to be dismantled to repair the plinth. So after the repair the house has to be reconstructed over it. The lower angles designed in the roof slopes would reduce wind load. As the house structures have become smaller in size, the extension of the eaves of the roof had to be trimmed down to reduce the wind pressure. This in turn makes the mud plinth susceptible to rain damage. It was found that the C I sheet roof was more suitable for collecting rain water and the inhabitants were using this benefit.

Houses with verandahs were appreciated well as the space could be used for domestic chores and keeping goats and chickens at night. The entry steps were appreciated as according to the users it gave a character to the house. Distinct entry steps in the front are a feature and part of aspiration of the users in this region. Platforms overhead gave them extra space to store.  This feature is present in the traditional houses too, so the users felt connected with these quality spaces.

The recipients of these houses were females. Perhaps this practice is gradually bringing changes in the social system and helping them to be decision makers in the family which may gradually bring changes in the habitation planning.

But the drawbacks were that these relief houses were one room house irrespective of family size or need. Ventilation and lighting were not given much priority; many houses had openings only in the front. This created problems in circulation. Orientation within the site and surrounding was not considered while the houses were put up. They were generally oriented to the east or south in consideration of the macro climate. Functional outdoor spaces in the homestead plan relating to the main house were also not considered, e.g. placement of kitchen, storage for firewood, toilet, place to keep domestic animals inside the yard, play area of young children under adult supervision.

Many houses did not have verandas suitable for domestic purposes. The verandas provided did not adequately connect this multipurpose space with the other functional spaces. The head height of the veranda was not enough to allow a person to enter the house with a sack on his head. This created difficulties in bringing rice sacks inside as rice is stored on the platform for year round consumption. The dwellers of these houses did not own enough agricultural land and the overhead platform served to store the harvest from share cropping. Furthermore, the users felt that if the veranda was wider, the functional quality of the space would have been better, e.g. if a person is offering his prayer (namaaz) in the veranda, another person cannot pass as all the width space is taken for that particular function. The occupants as receivers of the houses had to compromise these primary and regular living spaces.

Though the C I sheet roofs of the relief houses were suitable for collecting rain water (especially important as drinking water is an acute problem here), none of the houses provided this service as a built-in approach. Some of the users customized the houses for this function themselves.

The costs associated with the construction could not be judged as different approaches were used by the different organizations. One organization hired craftsmen as contractual employees for the project; one implemented the job through local contractors and another one encouraged self help approach. A unit cost analysis was not possible for this and also for the options and qualities of materials, e.g. gradation of CI sheet, amount of brick and cement used etc.   

**Figure:**
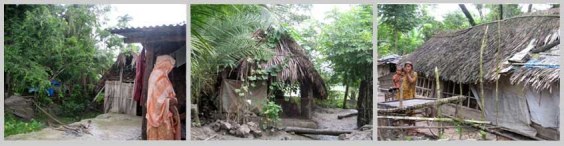
*Figure 10: Kitchen and hen coops constructed on self help*

The relief houses had potentials to create more positive spaces if the users were allowed to add more elements like another verandah, semi covered room, fences in the verandah. But they were not allowed so by the development organizations for evaluation and monitoring purpose. So it was found that the recipients were more interested in building their kitchens, chicken coops, cow sheds and other ancillary spaces in the homestead with much care that would be used for domestic and commercial purposes. They could personalize these and implement their ideas. Materials were recycled there, often from the damaged structures. The statement of Turner is found to be reflected here “*…the vital aspects of housing are not quantifiable at all. The most important “product” of any human activity is, of course, the satisfaction or frustration of needs*” (Turner, J, F, C, 1972)[17]. It seemed from the house construction activities triggered by the outside help that the inhabitants were compelled to satisfy this need through these ancillary constructions. But they could apply this energy in their house or the basic structure construction and thus participate in the building and dwelling process.

Though it was decided on the Shelter Recovery Strategy that community involvement would be ensured in decision making and actions, the full benefit of it could not be generated in the outcome in the field.

The dwellers were more familiar with the local haat unit in their construction and space use. Haat refers to the length from the tip of the middle finger to the elbow of an adult, i.e. eighteen inches. Constructions of dwelling spaces are practiced in an anthropomorphic sense and the users dwell in it in the same manner. The users felt unaccustomed with the practice of feet and inches in the inner spaces of the house as it did not match with their practice and perception.

Furthermore as the development organizations were assigned to deliver their “house” products in the particular areas, the visual planning developed like a colony or housing scheme that failed to produce a character to the place. The inhabitants needed to cultivate the flavor from the organic qualities of the place that would reflect the personal needs and aspirations, the built environment and the nature. The materials especially the new shiny C I sheet seemed like a disturbance among the green environmental setting and the visual scenario from its materialistic qualities.

Though the house relief of DanChurchAid did not have a model house and the users had freedom to design and construct houses on their own according to needs; the constructions often became very ambitious. As there were no guidelines, it could be apprehended from some constructions that it may not be possible to finish the work in the fixed budget. So the intention to let people decide and build on their own could not be fully utilized. Furthermore, there were deficiencies of low cost techniques.    Relief houses were given to only those who had their own lands. On the contrary, DanChurchAid grant was given to the landless. These people were making houses on government land.  This approach was courageous, one that needs more attention to reduce vulnerability of the landless. But the fear of eviction and thus sustainability of the program remained a question.

## Looking from Habitation and Dwelling Sense

*"Things which, as locations, allow a site we now in anticipation call building. They are so called because they are made by a process of building construction. Of what sort this making - building - must be, however, we find out only after we have first given thought to the nature of those things which of themselves require building as the process by which they are made* *" *(Heidegger, 1971)[1].

 The extreme poor are the more connected group with the nature in the habitation. Continuity with the changes of nature and thus staying sustainable is the character of their lifestyle (Kabir, K, H and Mallick, F, H, 2005)[18]. They are also the recipients of relief items after disasters. Outside interventions through disasters shape their lives more than the other inhabitants in a locality. So we find two opposite forces influencing the changes – nature with its available resources through the continuous span of time, and outside intervention with alien and often unaffordable supplies in short interrupted spans.

The dialogues between the relief houses and spaces in and around the homestead and the neighborhood failed to create the distinct colloquy of the locality. The houses failed to merge with the landscape and missed the identity of a distinct place, as if it could be placed anywhere but still remain alien.

The lifespan of the different age groups, their values and aspirations, culture and practices develop from the habitation. Perhaps it poses a question how the inhabitants in this locality as hunters (as fishermen) and gatherers (as forest material gatherers) adapt with these forces in the habitation. In this sense they need to continuously adjust with the “dwelling” quality in their territory. Again, to build one’s own house and provide it with the qualities of his “home” is perhaps an instinct both from male and female mind sets and activities, and both from the construction and design perspectives. It seemed that the inhabitants had to compromise this quality as a result of the outside interventions.

On this regard another approach that was carried out relating relief houses may be mentioned here. It was a participatory workshop organized by the Department of Architecture, BRAC University and BRAC for the cyclone Aila affected community people of Adarsha Gram of Padmapukur, Shyamnagar union, Shatkhira district during November, 2010. The inhabitants were landless people and hence living in this Adarsha Gram, a resettlement scheme provided by the government in the 1990s. The area was surrounded by swamps for artificial shrimp cultivation, lacked large trees as wind barriers and had been suffering from the damages from tidal actions, heavy erosion and salinity for about one and a half year since another cyclone, cyclone Aila of 2009.

**Figure:**
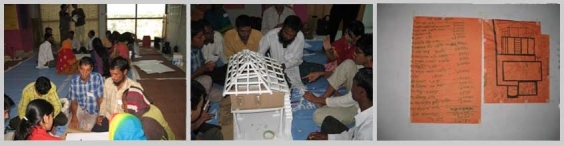
*Fig11: Participatory workshop for the community of Adarsha Gram*

The workshop aimed to blend the indigenous and formal knowledge to improve qualities considering house, homestead and habitation so the dwellers could have their share in rebuilding it with the development team. Carpenters, masons, house owners, community leader, people with knowledge on earth work and on local plants from the affected area and architects and engineers took part in the workshop. That the flavor of the habitation is maintained, local materials and techniques was a concern here. This was also favored for the limits of material options for the remoteness of the area and transport difficulties, compatibility against salinity, budget constrain and the apprehension of difficulties in construction for the unfriendly weather.

During the initial phase of the project the development team visited the site to have an understanding of the resources and constrains, organized focus group discussions and interviews of the inhabitants. This was followed by some more visits with a workshop in the site where the inhabitants made models of their “dream houses”. The aspirations of the house owners were reflected on this brainstorming session, where adaptability and wise use of the limited local resources were also echoed. Taking the practical points from this workshop the second workshop mentioned above was organized to blend the ideas more rationally with engineering knowledge and budget concerns. Two design options came out from these experiences gathered. Later only one option was chosen by the community to be implemented and they had the freedom to customize it according to the position of their site. The merits and gaps of this approach in sustainability and aspiration may be measured hopefully after the completion of the project.

## Conclusion

 Settlements are recreated to a degree after every major disaster. The physical and environmental characters are rectified in this sense to get adaptive with nature and sustainable with time. Perhaps this falls among the survival techniques.

Houses are one of the major elements of a settlement, with roads, links, growth centers, agricultural lands, water bodies, habitable and non- habitable areas. But houses as units indeed give a character to a settlement. If a house cannot meet the basic needs to function, it may be abandoned later and be replaced by another house that can reflect the needs and aspirations of the owner better. This may be built by the inhabitants when they reach the position to make houses on their own. From the housing practice in the rural areas, we find that usually the homesteads do not move but houses may need to be shifted to suitable sites, especially where there is scarcity of land and constrains in land ownership. In counties like Bangladesh with overwhelming population considering limited land, relocation after disasters is more often not possible. As many organizations provide housing reliefs after disasters, the ultra poor that suffer more in disasters receive this relief. But when they are not allowed to alter this ‘product” according to their needs and aspirations, they do not feel attachment with it as their homes. This was found from field visits in other different places that had also suffered from cyclone Sidr where some other organizations provided housing.

From the present practice of distribution of relief houses that lack attachments of the owners either in information sharing or building process and from the perception of dwelling, the sustainability of the relief houses may pose question of compatibility. There may be apprehension that the houses may only be used as shelters, like a temporary or transitional one to sustain life in the disaster phase, but these will lack the quality to be used as “houses” in the normal time phase.

## Funding

The study was done in 2008 for the thesis dissertation for Masters in Disaster Management at Postgraduate Programs in Disaster Management, BRAC University and was funded by Oxfam GB. The project concerning Adarsha Gram at Shatkhira was done in 2010 and was funded by UNDP. 

## Competitive Interest

 The author has declared that no competitive interests exist. 
